# IL-4, IL-10, CCL2 and TGF-β as potential biomarkers for severity in *Plasmodium vivax* malaria

**DOI:** 10.1371/journal.pntd.0010798

**Published:** 2022-09-30

**Authors:** Catalina Tovar Acero, Javier Ramírez-Montoya, María Camila Velasco, Paula A. Avilés-Vergara, Dina Ricardo-Caldera, Miquel Duran-Frigola, Gustavo Quintero, Myriam Elena Cantero, Juan Rivera-Correa, Ana Rodriguez, María Fernanda Yasnot-Acosta

**Affiliations:** 1 Grupo Investigaciones Microbiológicas y Biomédicas de Córdoba, GIMBIC, Universidad de Córdoba, Montería, Córdoba, Colombia; 2 Grupo de Enfermedades Tropicales y Resistencia Bacteriana, Universidad del Sinú, Montería, Córdoba, Colombia; 3 Doctorado de Medicina Tropical, SUE Caribe, Universidad de Cartagena, Bolívar, Colombia; 4 Grupo de Investigación en Estadística, Universidad de Córdoba, Montería, Córdoba, Colombia; 5 Ersilia Open Source Initiative, Cambridge, England, United Kingdom; 6 New York University School of Medicine, New York, New York, United States of America; Swiss Tropical and Public Health Institute: Schweizerisches Tropen- und Public Health-Institut, SWITZERLAND

## Abstract

Cytokines and chemokines are immune response molecules that display diverse functions, such as inflammation and immune regulation. In *Plasmodium vivax* infections, the uncontrolled production of these molecules is thought to contribute to pathogenesis and has been proposed as a possible predictor for disease complications. The objective of this study was to evaluate the cytokine profile of *P*. *vivax* malaria patients with different clinical outcomes to identify possible immune biomarkers for severe *P*. *vivax* malaria. The study included patients with non-severe (n = 56), or severe (n = 50) *P*. *vivax* malaria and healthy controls (n = 50). Patient plasma concentrations of IL-4, IL-2, CXCL10, IL-1β, TNF-α, CCL2, IL-17A, IL-6, IL-10, IFN-γ, IL-12p70, CXCL8 and active TGF-β1 were determined through flow cytometry. The levels of several cytokines and chemokines, CXCL10, IL-10, IL-6, IL-4, CCL2 and IFN-γ were found to be significantly higher in severe, compared to non-severe *P*. *vivax* malaria patients. Severe thrombocytopenia was positively correlated with IL-4, CXCL10, IL-6, IL-10 and IFN-γ levels, renal dysfunction was related to an increase in IL-2, IL-1β, IL-17A and IL-8, and hepatic impairment with CXCL10, MCP-1, IL-6 and IFN-γ. A Lasso regression model suggests that IL-4, IL-10, CCL2 and TGF-β might be developed as biomarkers for severity in *P*. *vivax* malaria. Severe *P*. *vivax* malaria patients present specific cytokine and chemokine profiles that are different from non-severe patients and that could potentially be developed as biomarkers for disease severity.

## Author summary

*Plasmodium vivax* is one of the main species responsible for malaria in the world. The pathogenic mechanisms leading to the development of severe *P. vivax* malaria are not yet fully understood. Immune system molecules such as cytokines and chemokines actively participate in the control of the infection, however, their uncontrolled production can influence alterations in organs such as the liver, kidneys, among others. In this study we show that there is a differential concentration of some cytokines and chemokines between patients with non-severe malaria and severe *P. vivax* malaria; and that there are associations between these molecules with manifestations that occur in severe malaria. Four molecules with potential to become biomarkers of severity were identified.

## Introduction

Malaria caused by *Plasmodium vivax* infection is prevalent in Southeast Asia and South America [1]. In the Americas region, it was responsible for 76% of malaria cases that occurred in 2019 [2]. Until recently, severe malaria was exclusively attributed to *P*. *falciparum*, but in the last decade, reports of severe cases caused by *P*. *vivax*, and to a lesser extent by *P*. *knowlesi*, have increased [3]. Severe clinical manifestations described for *P*. *vivax* infections include neurological conditions, especially coma or successive seizures, and impaired consciousness; hematological conditions, in particular anemia, severe thrombocytopenia and hemoglobinuria; systemic symptoms, such as circulatory collapse, vital organ damage, including respiratory dysfunction and acute respiratory distress syndrome, acute kidney failure, splenic rupture, liver dysfunction, and jaundice [4–6].

The pathophysiological mechanisms underlying *P*. *vivax* severe clinical manifestations are not clearly understood. It is believed that they are closely associated with the host’s immune response and are mediated by the activation of a pro-inflammatory response and increased expression of adhesion molecules in infected erythrocytes [7]. These would favor the pathophysiological process that triggers endothelial dysfunction and cell sequestration, generating microvascular flow obstruction and progressive deterioration of cellular metabolic processes [8]. In addition to the research interest of these molecules and their relationship to the pathogenic mechanisms involved in the disease, their study as potential biomarkers for severity in different pathologies have aroused great interest in recent years [9–11]. A biomarker for severity of *P*. *vivax* malaria that demonstrates predictive capacity in a longitudinal study would improve the diagnosis, classification, prediction, prognosis and follow-up for patients [12, 13].

Several cytokines, such as IL-6, IL-17, IL-12p40, TNF, CXCL10, IL-10 and IFN-γ, have been reported in higher concentrations in patients with acute *P*. *vivax* infections when compared to healthy controls and recovered patients [14]. Comparison of severe and non-severe *P*. *vivax* infections has identified that TNF-α, IL-10 and IL-6 levels are higher in severe patients [15,16]. Also, a positive correlation of cytokines, such as IL-10, IL-12 and IL-6, and parasite load has been observed in *P*. *vivax* infections [17,18], while a negative correlation was found between hemoglobin and IL-10, CXCL10 and IFN-γ levels [19]. Other clinical parameters, such as elevated creatinine, which is indicative of impaired kidney function, correlate with IFN-γ/IL-10 ratio in *P*. *vivax* malaria [20]. Likewise, higher concentrations of IL-10 were associated with complications in pregnant women with *P*. *vivax* malaria [21]. The identification of reliable biomarkers requires substantial evidence of their usefulness and efficiency under different epidemiological conditions before it can be generalized as a tool that supports the patient’s clinical management. The objective of this study is focused on identifying potential reliable biomarkers of severity in *P*. *vivax* infections by evaluating the cytokine and chemokine profile in patients with different clinical status in a low-endemicity epidemiological area.

## Methodology

### Ethical considerations

Participation in the study was voluntary, each individual signed an written informed consent or assent according to age; for child participants, formal parental/guardian consent was obtained.The project procedures were carried out in accordance with Resolution No. 008430 from October 4^th^, 1993, Republic of Colombia, Ministry of Health and the Helsinki Declaration and its amendments, the World Medical Association (WMA, Edinburgh, Scotland, October 2000). The Human Ethics Committee from the Health Sciences Faculty of Universidad de Córdoba granted approval for the study development, Act 001 of 2016.

### Study participants

One hundred and fifty-six (156) participants were enrolled, classified into three groups: patients with severe malaria (SM) (n = 50), non-severe malaria (NSM, n = 56) and healthy controls (HC) from endemic area (n = 50). Participants were recruited at the third level Hospital San Jerónimo, located in the Montería, the capital of the department of Córdoba, and at the first level Hospital San José de Tierralta Hospital. located in the urban capital of the municipality of Tierralta in the department of Córdoba between October 2017 and March 2019. Córdoba is a malaria endemic department in the north of Colombia, historically among the top five departments with the highest prevalence, accounting for an average of 20% of malaria cases in the country. Within the department, the municipality of Tierralta is the main endemic area for malaria transmission. After confirming the diagnosis of *P*. *vivax* malaria by microscopy and molecular techniques; biochemical, hematological and parasitological parameters were determined, clinical manifestations were reviewed in the clinical history and confirmed during the filling out of the epidemiological record from which, according to the World Health Organization (WHO) and the Colombian INS (National Health Institute, per its acronym in Spanish) recommendations [22,23] the patients were categorized as severe or non-severe malaria. Patients were classified as severe malaria if they met the criteria for any of the following complications: Hemoglobin concentration lower than 7 mg/dl was considered severe anemia, platelet concentration lower than 50.000 platelets/μl was considered severe thrombocytopenia, hypoglycemia (glucose < 60mg/dL), creatinine concentration higher than 1.3 mg/dl was considered indicative of renal dysfunction, Glutamic-pyruvic transaminase (GPT), Glutamic-oxaloacetic transaminase (GOT). concentration higher than 40 u/L was considered hepatic dysfunction. Epidemiological and clinical data were recorded in a survey. Healthy individuals were recruited among people accompanying the recruited *P*. *vivax* patients at the hospital. All individuals accompanying patients recruited in the study were offered to join the study as negative controls. These individuals joined the study voluntarily, were checked for clinical malaria symptoms at the time of recruitment, were confirmed negative by PCR for Plasmodium infection and were subjected to all the same laboratory analysis as the patients. Children under 2 years old, women in pregnancy, people with underlying diseases, mixed malaria, *P. falciparum* monoinfections, leptospirosis and dengue were excluded from the study for all groups.

### Determination of parasitological, hematological, and biochemical parameters

Blood samples were taken from each patient by venipuncture in EDTA, for hemogram, parasitological tests, and plasma collection, and dry tubes (no anticoagulant), for quantification of biochemical parameters. These were obtained during the febrile period and before starting the antimalarial treatment. The following procedures were carried out on the samples: automated blood cell count, thick blood smear, peripheral blood smear, serum quantification of glucose, creatinine, total bilirubin, direct bilirubin, aspartate aminotransferase, alanine aminotransferase and monoinfection confirmation by nested polymerase chain reaction (PCR) [24].

### Cytokines and chemokines plasma quantification

IL-4, IL-2, CXCL10 (IP-10), IL-1β, TNF-α, CCL2 (MCP-1), IL-17A, IL-6, IL-10, IFN-γ, IL-12p70, CXCL8 (IL-8), and active TGF-β1 determination was performed in plasma, using the Human Essential Immune Response Panel kit (13-plex) (Catalogue. No. 740930) from Biolegend.

The procedure was carried out in accordance with the manufacturer’s recommendations using equal amounts of plasma (10 μL) and beads (10 μL) Samples were run in duplicates in a FACSCalibur (Becton Dickinson, Franklin Lakes, NJ).

### Statistical methods and variables

Comparisons between groups were analyzed using the Kruskal-Wallis and the Mann-Whitney tests, according to the number of groups to be analyzed. P-values less than 0.05 were considered significant. Correlation between variables were performed using Spearman’s rank correlation. R software was used for graphs and statistical analysis.

Association between of cytokines and chemokines with the clinical outcome of *P*. *vivax* patients was determined by Generalized additive model for location, scale and shape (GAMLSS) [25]. The dependent variable was the clinical outcome (severe/uncomplicated), and the independent variables were: IL-4, IL-2, CXCL10, IL-1β, TNF-α, CCL2, IL-17A, IL-6, IL-10, IFN-γ, IL-12p70, CXCL8 and active TGF-β1. The variables were selected based on a 5% individual significance test for the final Gamlss model after a preliminary selection of the Lasso regression method. The area under the curve (AUC) of the receiver operating characteristic (ROC) curve assessed the model performance. Measures of PPV, NPV, specificity, sensitivity related to the confusion matrix were additionally calculated considering group test partitioning (20%) and group training (80%). Performance reported in this study corresponds to the test set.

## Results

### Clinical characteristics of *P*. *vivax* malaria patients

**Fig 1:**
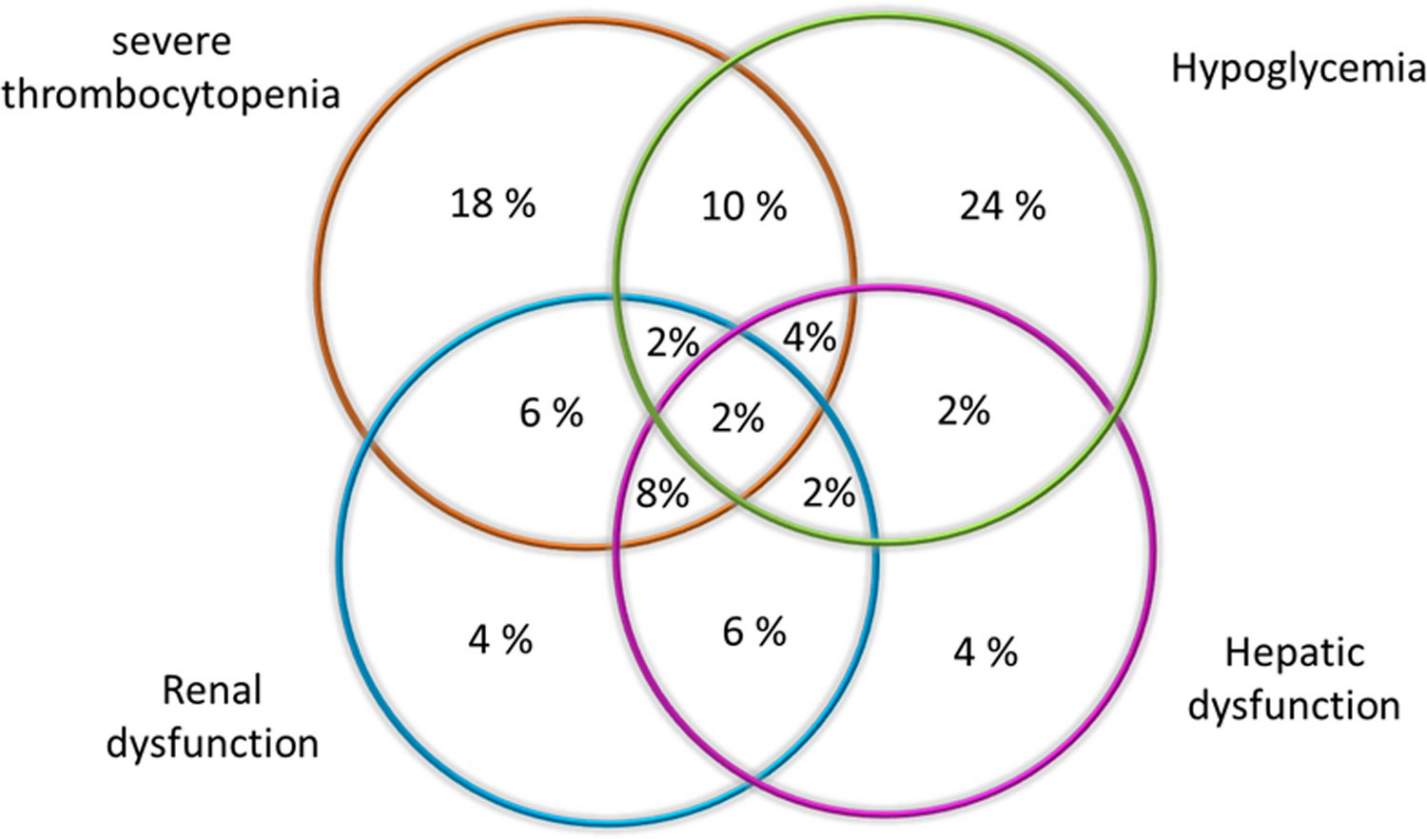
Venn diagram showing the frequency of manifestations and their combinations in the individuals with severe malaria (N = 50).

One hundred and fifty-six individuals were enrolled in the study, with 80 men and 76 women in an age range between 3 and 71 years old; participation in the study was voluntary, each individual signed an informed consent or assent according to age. The age was similar in each of the study groups: healthy, *P*. *vivax* non-severe and severe infections (*p* = 0.57, Kruskal-Wallis test). The most frequent clinical manifestations in patients with non-severe malaria were fever, headache and chills. In the group of patients with severe malaria, the main findings are illustrated in Fig 1. The patients in the study did not present cerebral malaria or pulmonary alterations. The clinical and epidemiological characteristics of this cohort were previously described [26].

### Cytokines relation to clinical outcome in non-severe and severe patients with *P*. *vivax* infections

**Fig 2:**
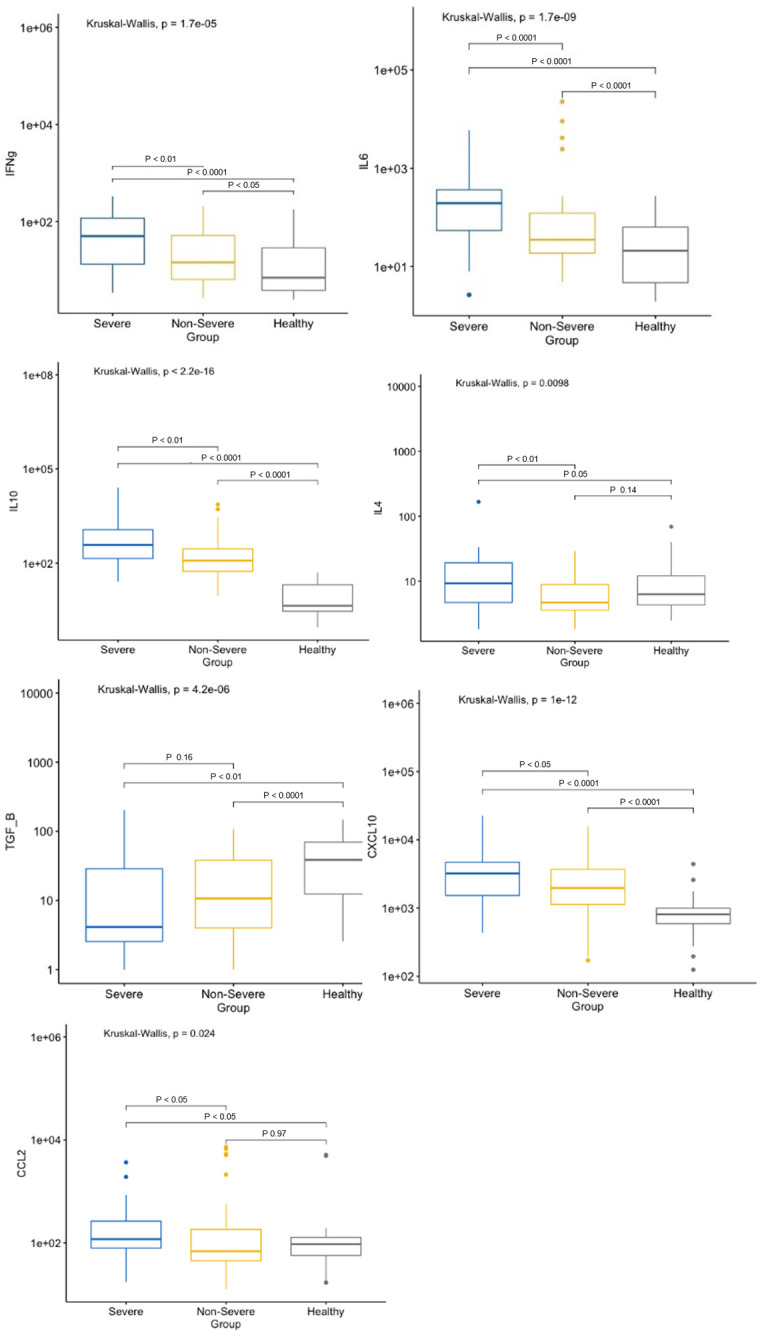
Variation of cytokine and chemokine concentrations in patients with severe and non-severe *P*. *vivax* malaria. Severe (n = 50), Non-severe (n = 56), Control Healthy (n = 50). Comparisons between groups were performed using the Mann Whitney test. Concentrations in pg/ml.

Plasma concentrations of a panel of cytokines and chemokines were determined in 156 plasma samples from patients with severe (n = 50) and non-severe (n = 56) *P*. *vivax* malaria, as well as from healthy controls (n = 50). The median concentration was significantly different between the non-severe and severe groups for IL-4, CXCL10, CCL2, IL-6, IL-10, IFN-γ and active TGF-β1 (Fig 2). However, no differences were found between groups for IL-2, CXCL10, IL-1β, TNF-α, IL-17A, IL-12p70 and CXCL8. Among cytokines that were different between groups, only TGF-β1, presented a higher concentration in healthy controls than in patients with severe and non-severe disease (Fig 2).

### Association of clinical and immunological variables in *P*. *vivax* malaria patients

**Fig 3:**
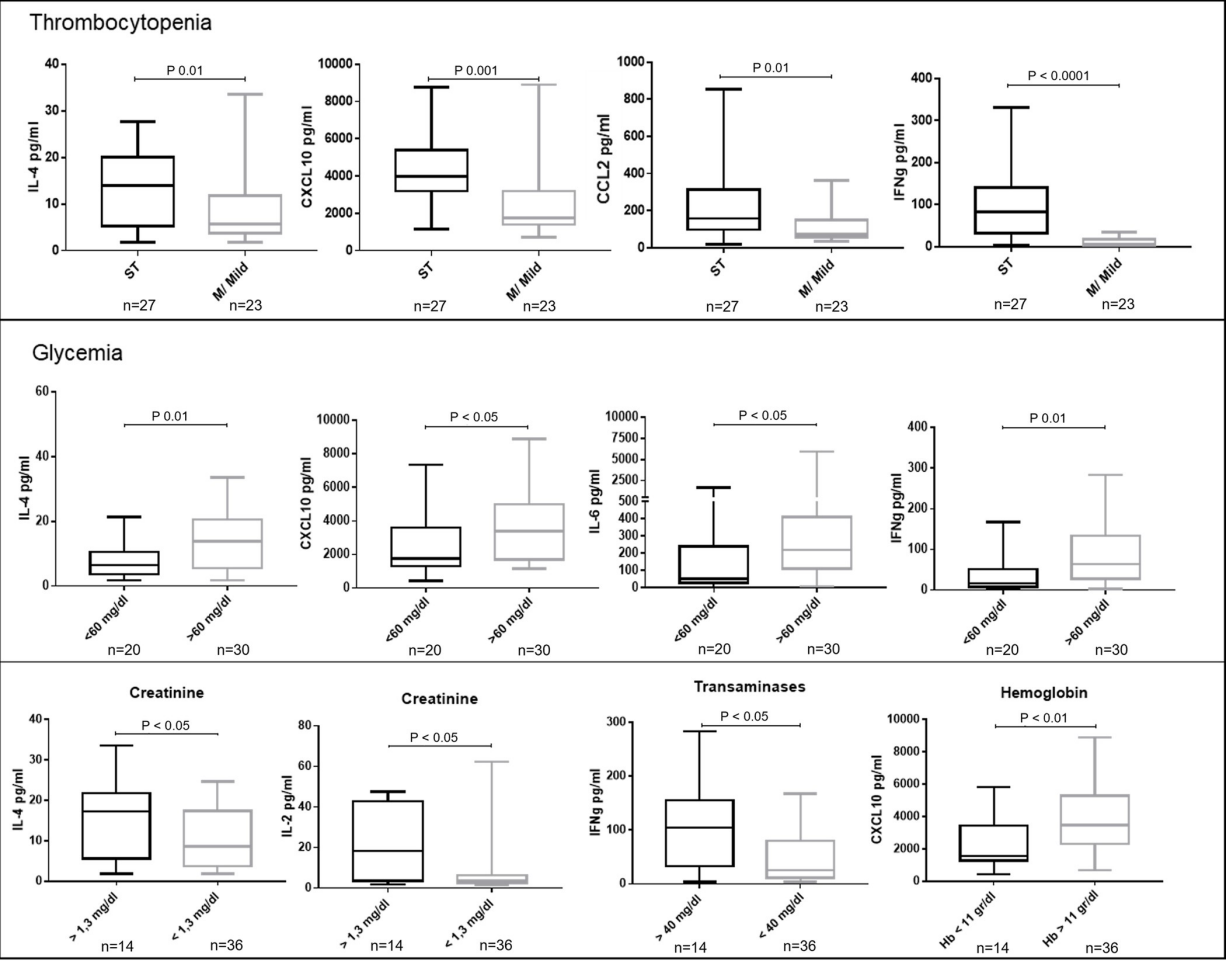
Cytokine concentrations in patients with different laboratory parameters that determine severity in *P*. *vivax* malaria. Severe thrombocytopenia (ST) (platelets ≤ 50,000/uL), mild/moderate thrombocytopenia (M/Mild) (50,000–150,000 platelets/uL). Glutamic-oxaloacetic transaminase (GOT) / Glutamic-pyruvic transaminase (GPT) above 40 U/L. The graphis in this figure include only patients with severe malaria. No significant differences were found in the levels of other cytokines (panel described in Table 1) not included in the figure.

Clinical laboratory parameters of *P*. *vivax* malaria patients were determined in plasma samples obtained at the time of diagnosis. The relationship of cytokine and chemokine levels with clinical laboratory parameters was analyzed in severe malaria patients to determine whether the levels of specific cytokines or chemokines were associated with different malaria complications. Clinical parameters were divided into groups according to the laboratory criteria established by WHO and INS for malaria patients. These analyses showed that IL-4, CXCL10 and IFN-γ levels were significantly higher in patients with severe thrombocytopenia or with hypoglycemia. It is important to note that the sample size in some of the groups of these analysis is low, and interpretation of these associations should be considered with caution. Other parameters were found to be associated with different cytokines, such as low hemoglobin with CXCL10, creatinine with IL-4 or transaminases with IFN-γ (Table 1 and Fig 3). Taken together, these data suggest that different malaria complications are associated with specific cytokines in this cohort.

**Table 1:** Cytokine and chemokine concentration by laboratory parameters in the entire study cohort population. The median and interquartile ranges of the molecules are shown. Concentration in pg/ml. Total subjects (n = 156), severe malaria (n = 50), non-severe malaria (n = 56), healthy controls (n = 50).*Only infected subjects (n = 106).

	IL4	IL2	CXCL10	IL1B	TNFα	CCL2	IL17A	IL6	IL10	IFNγ	IL12p70	CXCL8	TGFβ
Parasite count/μL*													
<5,000 (n = 82)	6.2 (3.7–17.3)	3.6 (2.7–35)	2168 (1267–3961)	9.9 (5.4–36.0)	4.1 (2.9–15.8)	95 (54.1–192)	3.7 (2.1–19.5)	80.2(22.0–183)	155 (66.2–523.2)	22.1 (7.5–78)	2.6 (2.2–30)	14 (6.0–33)	10.7 (2.6–40)
5,000–10,000 (n = 16)	6.8 (5.3–20.6)	3.6 (2.2–3.7)	3921 (2115–5443)	10.2 (6–18.4)	3.9 (3.3–14.8)	100 (45.5–470)	3.4 (2.0–4.7)	69.9 (29.6–508)	844.5 (106–1364)	43 (14.7–104)	2.7 (2.6–5.5)	8.6 (5.9–24.5)	11.6 (4.1–41)
> 10,000 (n = 8)	5.8 (4.2–11.4)	3.3 (2.0–3.6)	2864 (1623–6358)	7.9 (2.6–6)	3.6 (2.6–6)	221 (101–482)	3.5 (2.1–5.6)	345 (144–1301)	722.3 (247–1154)	44.5 (16–135)	2.6 (2.5–3.2)	9.1 (5.3–12.9)	2.6 (1–10.2)
**Hemoglobin g/Dl**													
<12 (n = 85)	6.6 (4.4–17.3)	3.6 (2.6–36)	1530 (893–3479)	11.3 (6–34.2)	3.9 (2.9–12.6)	90.1 (52.8–164)	3.9 (2.1–20)	70 (21.5–180)	110 (27.3–404)	14.2 (6–70.2)	2.7 (2.3–25)	13 (7–36.4)	12.3 (3.5–44)
≥12 (n = 71)	6.5 (3.8–16)	3.6 (2.9–27.1)	1296 (850.3–3231)	13 (6.3–34)	3.9 (3.0–6.8)	103 (56–188)	4.7 (2.2–20)	47.1 (11–136)	37 (8.2–338)	20.3 (4.7–56)	2.6 (2.1–19)	17 (7–29)	25 (4.1–48)
**Platelets/μL**													
≤ 50,000 (n = 27)	14 (5.6–19.7)	3.6 (3.5–38)	4027 (3217–5443)	16 (6.7–40)	6.6 (3.9–18)	157 (97.4–312)	6.5 (3.4–20)	224 (118–443)	651 (154.3–1614)	82 (33–141)	3.3 (2.6–38)	17 (9–36.1)	8.1 (2.6–41)
50,000 <100,000 (n = 34)	5.3 (3.7–14)	3.6 /2.3–3.7)	1650 (1318–3079)	6.5 (4.5–23.1)	3.9 (2.8–7.3)	80 (53–189)	3.1 (2.1–10.4)	95.2 (30.8–246)	218.9 (92–897)	22.1 (8.3–63)	2.6 (2.0–3.5)	8.7 (5.2–27)	4.1 (1.8–27)
100.000–150,000 (n = 23)	5.7 (3.7–10.6)	3.6 (2.3–27)	2387 (1560–4230)	12 (5.5–30.8)	4.1 (2.9–6.3)	81 (53–195)	3.2 (2.1–16)	37.7 (18–85)	107 (30–334)	14.2 (8.0–44)	2.6 (2.1–5.7)	15.7 (4–34)	20 (4.1–39)
≥150.000 (n = 72)	6.1 (4.2–15)	3.6 (2.9–26.2)	870 (621–1298)	14.1 (7.3–34)	3.9 (2.9–6.1)	90 (41–129.3)	4.5 (2.7–20)	20 (6.2–66)	20 (2.9–50.2)	7.7 (3.8–38)	2.6 (2.2–8.2)	17 (6.8–36)	33.4 (10.4–62)
**Glycemia mg/Dl**													
< 60 (n = 22)	7.5 (3.9–14)	3.6 (3.2–3.6)	1650 (1191–3569)	10 (4–18.2)	4.1 (3.8–7.0)	97 (58.9–236)	3.9 (2.1–9.8)	63 (31–304)	163 (69–1376)	17 (8.6–77.8)	2.6 (2.6–5.0)	10.8 (5.4–15)	3.6 (2.6–32)
≥ 60 (n = 134)	6.3 (4.2–16.5)	3.6 (2.9–33.4)	197 (844–3255)	13 (6.0–34.6)	3.9 (2.9–9.5)	96.4(53–176.4)	4.1 (2.1–20)	46 (15–136.4)	67 (11.6–284)	19 (5.1–61)	2.6 (2.2–21)	17.0 (7.0–36)	23.1 (4.1–46.5)
**Creatinine mg/Dl**													
< 1.3 (n = 138)	5.9 (3.9–13)	3.6 (2.8–24)	1391 (833–3130)	11.2 (5.7–32)	3.9 (2.9–7.0)	96 (52–181.1)	3.7 (2.1–20)	38.2 (14.6–138)	68.2 (16.7–284)	14.2 (5.1–50.3)	2.6 (2.2–6.1)	13.4 (6.4–30)	16.9 (4.1–45.3)
≥ 1.3 (n = 18)	20.7 (6.6–22)	29.9 (3.5–43)	2925 (125–4422)	26.8 (8.1–47)	11.7 (3.9–20)	100.4 (71.2–247)	19.5 (2.5–20)	130.8 (53.5–253)	157 (83.5–854)	68 (26–122.2)	29.4 (2.6–39)	28.1 (9.2–33)	23.9 (3.7–45)
**GOT U/L**													
< 40 (n = 141)	6.06 (4.1–15.6)	3.6 (2.8–28)	1341 (818–2901)	11.3 (5.8–34)	3.9 (2.9–7.1)	90 (52–162)	3.9 (2.1–20)	40.3 (16–127)	80.4 (17–271.3)	14 (5.2–50.2)	2.6 (2.2–12.3)	14.4 (6.6–32)	19 (4.1–47.2)
≥ 40 (n = 15)	50,8 (44,8–73.2)	6.7 (3.6–35.2)	3487 (2190–5926)	12,8(7.9–38)	12 (3.4–20)	187 (100–315)	12 (2.5–20)	261 (103.4–1587)	481 (75–3352)	104 (33–156)	3.2 (2.6–32.2)	25 (7–41)	17 (2.6–41.5)
**GPT U/L**													
< 40 (n = 126)	6,06 (4,0–15,6)	3,6 (2,8–28,0)	1341 (833–2914)	11,3 (5,7–33,3)	3,9 (2,9–6,9)	90,2 (52,6–162,4)	3,9 (2,1–19,5)	40,3 (16,2–124)	67,3 (15–278)	14 (5–50)	2,6 (2,1–15)	14,5 (6,6–31)	20,4 (4,1–49)
≥ 40 (n = 30)	8,4 (4,5–19,8)	3,6 (3,4–35)	2190 (1063–4462)	13 (7,5–37,1	3,9 (3,5–16)	119,2 (59–259,3)	5,3 (2,1–20)	134,4 (16–526)	175 (56,1–1005)	51,4 (9,5–122)	2,7 (2,6–31,1)	13,5 (6,5–40)	11,1 (2,6–36,2

**Fig 4:**
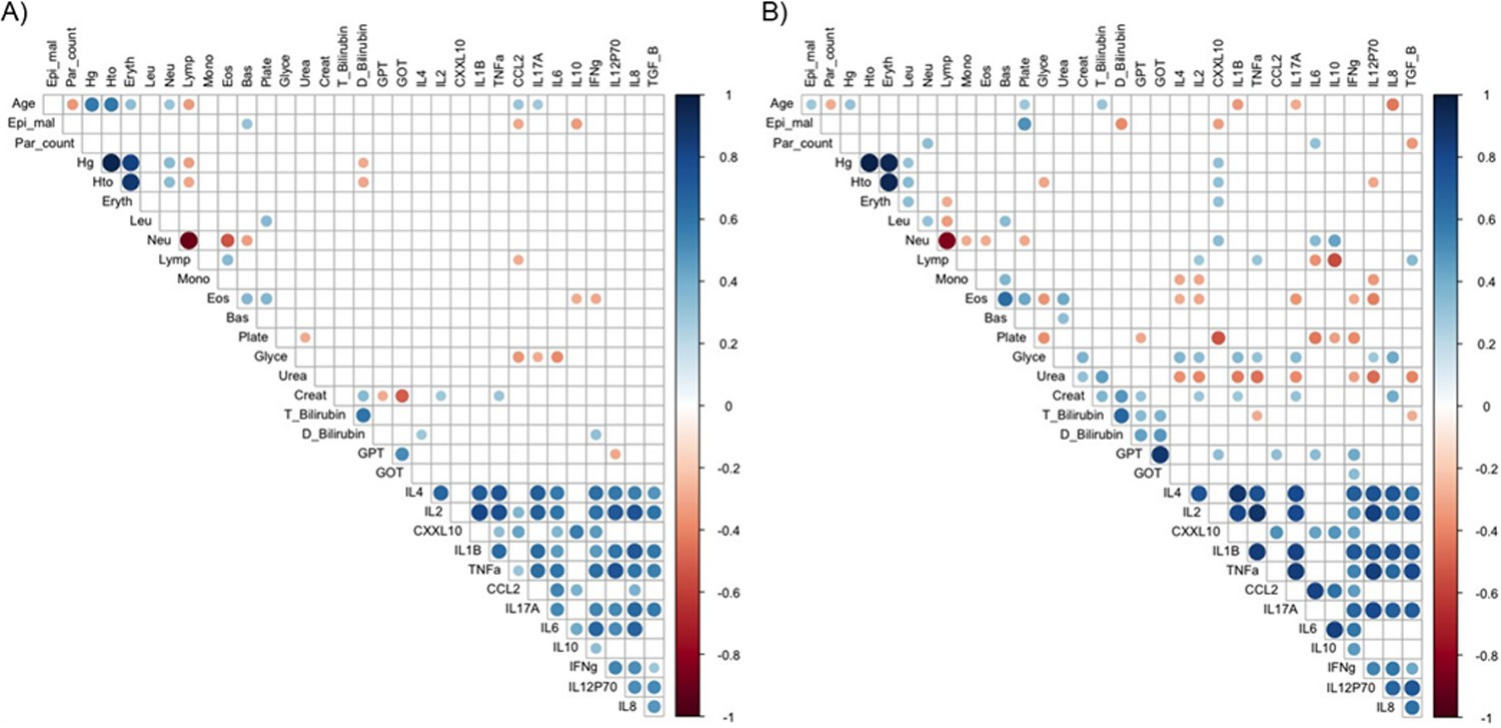
Correlation of clinical parameters and cytokine levels in patients with A) non-severe and B) severe *P*. *vivax* malaria. The spot at the intersections between the variables indicates a correlation with p <0.05. The size and color of the spot is proportional to the strength of the correlation, blue for positive correlations and red for negative correlations (scale on the right). Epi_mal, previous malaria episodies; Par_count; parasite count; Hg; hemoglobin, Hto; hematocrit; Eryth, erythrocytes; Leu, leukocytes; Neu, neutrophils; Lymp, Lymphocytes; Mono, monocytes; Eos, Eosinophils; Bas, Basophils; Plate, platelets; Gly, glycemia; Creat, creatinine; T_Bilirubin, Total bilirubin; D_Bilirubin, direct bilirubin; GPT, Glutamic-pyruvic transaminase; GOT Glutamic-oxaloacetic transaminase.

We then performed a general analysis to identify potential associations between the different cytokines and chemokines and clinical parameters in the severe and non-severe groups. Spearman’s analysis shows a positive correlation among many of the different pro-inflammatory and anti-inflammatory cytokines and chemokines, in both groups (Fig 4), which is characeristic of the ‘cytokine storm’ induced by malaria [27]. In general, severe patients present more significant correlations with different immune cell-types, which is expected considering the inflammatory response with higher levels of cytokines and chemokines in the plasma of severe patients, as observed in Fig 2.

A negative correlation between age and parasite count was also observed in both patient groups, which may be a consequence of a more effective immune response in older patients compared to children (Fig 4). In the severe malaria group, age has a positive relation with previous malaria episodes, but negative with parasite counts, probably as a consequence of previous infections that result in partial protection. Also, correlations with clinical parameters that define specific complications, such as creatinine and urea for renal disfunction, are more frequently observed in severe patients. These relations may not reflect a causative effect, but may be a consequence of the simultaneous elevation of cytokines and the parameters that define malaria complications in the severe group.

In patients with severe malaria, platelets showed negative correlations with CXCL10, IL-6, IL-10 and IFN-γ. 90% of these patients presented thrombocytopenia with different degrees of severity (mild, moderate, severe). For glycemic concentration, it was positively correlated with IL-4, IL-2, IL-1β, TNF-α, IL-17A, IL-12p70 and IL-8. Creatinine concentration is positively correlated with IL-2, IL-1β, IL-17A and IL-8. In the GOT case, it presented a positive correlation with CXCL10, CCL2, IL-6 and IFN-γ. This last cytokine was correlated in the same way with GPT. In patients with severe malaria, a slight decrease was observed in the number of correlations between pro-inflammatory and anti-inflammatory cytokines and chemokines; however, more moderate and strong correlations were observed when compared to the non-severe malaria group (Fig 4B)

### 3.5 Cytokines associated with severe *P*. *vivax* malaria

To find the combination of cytokines and chemokines that can better discriminate between patients with severe or non-severe *P*. *vivax* infections, the 13 quantified potential biomarkers were analyzed using the Gamlss Model in the confusion matrix of observed values, which showed that the joint analysis of the IL-4, IL-10, CCL2, and TGF-β shows significant differences between patients with severe and non-severe malaria due to *P*. *vivax*.

**Fig 5:**
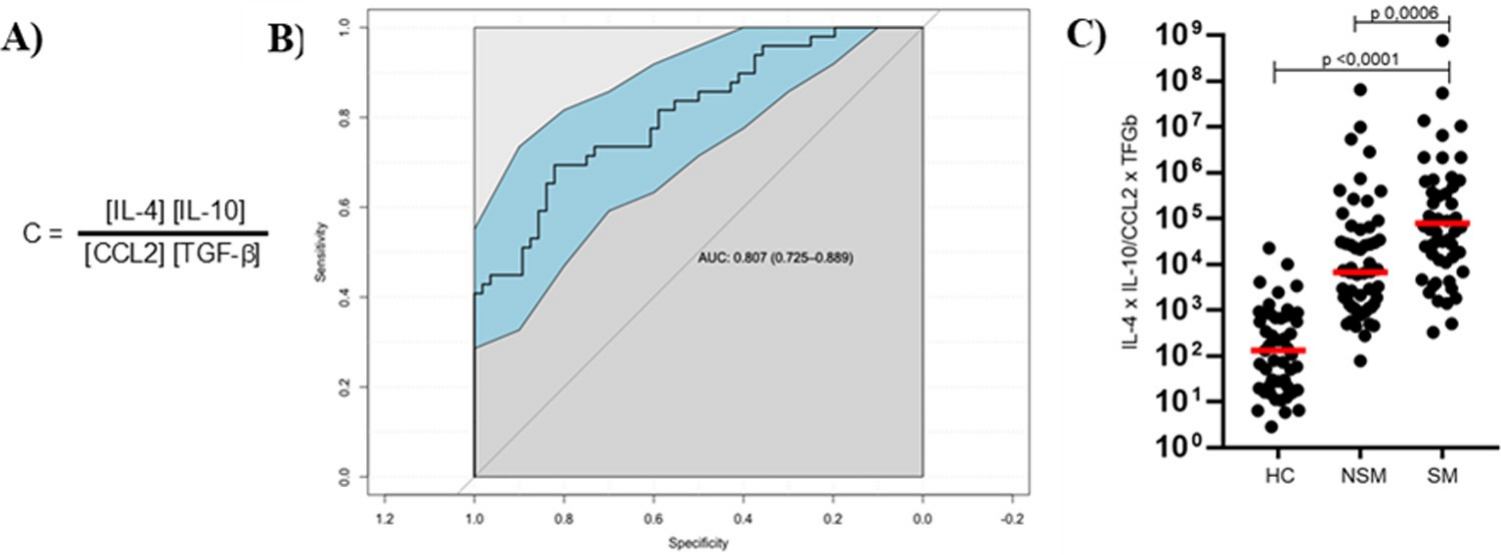
IL-4, IL-10, CCL2 and TGF-β as biomarkers for severity in *P*. *vivax* infections. A) Coefficient formula. B) ROC analysis showing the performance of IL-4, IL-10, CCL2 and TGF-β in distinguishing the severe malaria from non-severe malaria. C) Scatter plots showing C values. ROC, receiver operating characteristic curve; AUC, area under the curve; HC, Healthy controls; NSM, non-severe malaria; SM, severe malaria.

The ROC curve analysis yielded an area under the curve (AUC) of 80.7% with 95% confidence interval, showing that the Gamlss model is efficient to discriminate the clinical outcome of the patients, displaying the following values: 69% sensitivity, 75% specificity, a positive predictive value of 70%, and a negative predictive value of 73%. A coefficient (C) was calculated with the concentrations of cytokines and chemokines selected by the model, taking into consideration whether their levels increase or decrease in relation to controls (Fig 5). The value of the coefficient for each patient was significantly higher in the severe group compared with the non-severe group (Fig 5).

## Discussion

The main goal of this study was to determine the levels of a panel of cytokines and chemokines and test their effectiveness as a tool to distinguish between severe and non-severe *P*. *vivax* infections. The long-term objective is that once a *P*. *vivax* malaria diagnosis is confirmed in a patient, the clinical course could be predicted, that is, whether that patient will suffer from severe or non-severe malaria. The potential biomarkers identified in this study will have to be validated to assess whether they could predict the development of severe malaria before the symptoms associated with the complication become evident.

Thrombocytopenia was one of the main manifestations in individuals with *P*. *vivax* infections; as previously reported in other studies [28,29]. Platelet levels were negatively correlated with levels of CXCL10, a chemokine that is produced in response to IFN-γ, attracts immune cells to the inflammation focal point and its high concentration has been associated to severity in diseases with different etiology, including malaria, both in experimental models and in patients with *P*. *falciparum* infections [30]. Increased levels of CXCL10 and IFN-γ have been reported in severe thrombocytopenia caused by viral infections [31], and malaria [32]. Additionally, CXCL10 is associated with immune thrombocytopenia [33]

Naing *et al* reported that low IL-6 concentrations are associated with severe thrombocytopenia, but in our study a negative correlation was evidenced between these variables. Patients with severe thrombocytopenia had the highest IL-6 concentrations, compared to patients with moderate/mild thrombocytopenia. *In vitro* studies have shown that IL-6 stimulates megakaryocytes proliferation and maturation, resulting in an increase in platelets production. Our result could show a restoration mechanism of plasma platelets concentration by increasing IL-6 production [34].

IL-10 concentrations correlated negatively with platelet counts, which is similar to a previous study in *P*. *vivax* and *P*. *falciparum* infections [35]. IL-10 in high concentrations decreases pro-inflammatory cytokines production, which in turn is related to a decrease in platelet progenitor cells [36]. This can contribute to the development of severe thrombocytopenia. The increased IL-10 concentration in patients with severe malaria can be explained as a response to the elevated levels of pro-inflammatory cytokines [37]. In this study, a strong direct correlation was found in severe *P*. *vivax* patients between IL-10 and the pro-inflammatory IFN-γ and IL-6.

Renal dysfunction, based on serum creatinine increase, was associated with an increase in total bilirubin, direct bilirubin, GOT and with a pro-inflammatory cytokine profile including IL-2, IL-1β, IL-17A and IL-8. These results are similar to previous reports, such as the study published by Cruz and coworkers in the Brazilian Amazon, in which they found that the inflammatory ratio (IFN-γ/IL-10) was higher in patients with increased serum creatinine compared to those with normal creatinine [38]. Likewise, the study by Mendoҫa and coworkers also found a relationship between increased creatinine and IFN-γ [39]. Recruitment of inflammatory cells to the kidneys, such as neutrophils and macrophages that release pro-inflammatory cytokines, contribute to the activation of renal and tubular endothelial cells, generating alterations in the vascular, tubular and glomerular functions of the kidneys [40,41]. In this way, it is possible that pro-inflammatory cytokines in the patients in our study contributed to renal dysfunction.

In our study, patients with severe *P*. *vivax* infections showing liver abnormalities, showed a positive correlation between GOT and CXCL10, CCL2 and IL-6 concentrations, as well as between GOT and GPT with IFN-γ. Similarly, Yeom and coworkers previously reported the association of IL-4, IL-6, IL-10, TNF-α and IFN-γ in Asian patients with this same condition [42].

Hypoglycemia was related to low IL-4, CXCL10, IL-6, and IFN-γ concentrations, regarding patients with normal glycemia concentrations. These results are different from other studies, where increases in cytokines, such as IL-6, IL-1 and TNFα, were related to decreases in serum glycemia. Molecules such as CXCL10 and IL-6 have been reported to be involved in abnormal glucose metabolism by inhibition of hepatic gluconeogenesis decreasing the concentration of glycemia in the blood [43–45], which may explain our result in patients with hypoglycemia.

Several studies have been published evaluating differences in cytokines and chemokines concentrations between groups of patients with severe or non-severe *P. vivax* malaria, which allow us to analyze the behavior of these molecules in the different clinical outcomes for the disease. However, it is rare to find studies that use statistical models to analyze the relation of different cytokine combinations with the clinical outcome of *P*. *vivax* infections. If the results presented here are validated in a longitudinal analysis to demonstrate predictive value, this approach could constitute a useful tool when analyzing the general pathology scenario considering the biological interactions that result from communication between the parasite and the host.

In this study, a statistical model identified that the combined relation of increased IL-4 and IL-10, with decreased CCL2 and TGF-β levels, was associated with severity in *P*. *vivax* infections. The combination of biomarkers may be a better way to provide improved diagnostic accuracy than single markers. Previous studies had shown that IL-4 has a leading role in a network of interactions with other cytokines in patients with *P*. *vivax* malaria [39]. Additionally, it is known that IL-6, CCL2 and IL-10 could be recognized as biomarkers for *P*. *vivax* acute infection phase [14], however, our study is unique in proposing a coefficient combining levels of four cytokines and chemokines as biomarkers for *P*. *vivax* severity.

TGF-β is a regulatory cytokine; in this study, healthy controls had a higher concentration of this molecule when compared to malaria patients. Andrade *et al* reported a lower TGF-β concentration in patients with severe malaria compared to non-severe malaria [46]. In children with *P*. *vivax* malaria, decreased TGF-β concentrations have been related to severe anemia [47], highlighting its protective role against certain pathogenic manifestations, and its positive effect observed in the model.

High IL-4 concentrations in *P*. *falciparum* infections correlate with recovery and a decrease in parasite count in patients. Moreover, this cytokine concentration in pregnant women with *P*. *vivax* malaria is negatively correlated with birth weight; therefore, it has been proposed in previous studies as a prognosis for severity or poor prognosis during the clinical course [21,48]. In our study, IL-4 and IL-10 concentrations were higher in patients with severe malaria than in patients with non-severe malaria, consistent with that reported by other authors [49]; the increase in these cytokines may reflect the immune response controlling the infectious process [36,50].

Patients with severe *P*. *vivax* malaria present a pro-inflammatory/anti-inflammatory cytokine profile with elevated IFN-γ, IL4 and IL6/IL10, respectively. The chemokines CXCL10 and CCL2 were also elevated in severe patients. TGF-β appears to be suppressed, which may explain the high levels of pro-inflammatory cytokines in patients with severe *P*. *vivax* malaria. Conversely, TGF-β is elevated in non-severe malaria patients, suggesting that it contributes to an effective regulation of inflammation. Thrombocytopenia and hypoglycemia were the most characteristic manifestations in the severe *P*. *vivax* malaria group, having a shared pro-inflammatory cytokine/chemokine profile (IFN-γ, IL4, IL6 and CXCL10). Validation of these results in longitudinal studies may demonstrate that the joint analysis of IL-4, IL-10, CCL2 and TGF-β plasma concentrations could be useful to identify patients at risk of developing complications in *P*. *vivax* malaria.

Our long-term goal is to identify biomarkers that could predict the development of severe malaria before the symptoms associated with the complication become evident. In this way, timely clinical care can be provided to avoid the complication, improving clinical care and the quality of life of the patient.

Our study has some limitations. First, longitudinal samples from patients after infection but before they developed severe disease were not available; however, groups with different clinical conditions during acute phase were established in order to perform the analyses. Second, the sample size is small, but the number of individuals in each group allows statistical analysis. Increasing the number of individuals will allow us to strengthen the observations and broaden the analyses.
